# Supplementary material for: Cytosine base editor 4 but not adenine base editor generates off-target mutations in mouse embryos
Source: Commun Biol. 2020 Jan 9;3:19. doi: 10.1038/s42003-019-0745-3 (PMC6952419; doi:10.1038/s42003-019-0745-3)
Supplement: Supplementary file 1 — Supplementary Information [file 42003_2019_745_MOESM1_ESM.pdf]

```
      -36                                                              Frequency (%)
WT     CCCAGCAGGACATCTCTTCCTGCCCATGACACCCTTGGCACAGTATGGGCCCTTCTGGGAAGTG
ABE 1  .........................................G..G................... 49.5
       .......................................G.G..G................... 30.5
       .......................................G.G...................... 15.3

ABE 2  .........................................G..G................... 60.2
       .......................................G....G................... 18.4
       .......................................G.G..G................... 18.1

ABE 3  .......................................G.G..G................... 69.8
       .........................................G..G................... 27.0

ABE 4  .........................................G..G................... 89.6
       .......................................G.G...................... 6.4

ABE 5  .......................................G.G..G................... 57.6
       .........................................G..G................... 39.2

ABE 6  .......................................G.G..G................... 45.3
       .......................................G.G...................... 28.2
       .........................................G..G................... 23.3

ABE 7  .......................................G....G................... 36.8
       .......................................G.G..G................... 34.2
       .........................................G..G................... 24.8
                                               ^GCAC

ABE 8  .........................................G..G................... 92.5
       .........................................G...................... 5.8

ABE 9  .......................................G.G..G................... 45.9
       .......................................G.G...................... 39.0
       .......................................G....G................... 11.3

ABE 10 .......................................G.G..G................... 70.3
       .......................................G.G...................... 26.0

ABE 11 .........................................G..G................... 50.0
       .......................................G.G...................... 45.3

ABE 12 .......................................G.G...................... 44.0
       .........................................G..G................... 32.3
       .......................................G.G..G................... 19.5

ABE 13 .......................................G.G...................... 50.2
       .......................................G.G..G................... 37.8
       .......................................G....G................... 6.1
```

```
-36                                                               Frequency (%)
WT     CCCAGCAGGACATCTCTTCCTGCCCATGACACCCTTGGCACAGTATGGGCCCTTCTGGGAAGTG
BE4 1  ........................................T....................... 41.0
       ..--------------------------------------........................ 54.1

BE4 2  ...............T..T.....................T....................... 37.8
       ........................................G....................... 37.5
       ........................................A....................... 12.2

BE4 3  ........................................T....................... 53.4
       ...............A........................T....................... 46.6

BE4 4  ......................................T.T....................... 47.2

BE4 5  ........................................T....................... 62.9
       .............-.T..T.....................T....................... 37.1

BE4 6  ...............T......................T.T....................... 56.9
       .............-.T..T.....................T....................... 38.8

BE4 7  ...............T......................T.T....................... 42.8
       ........................................T....................... 6.3

BE4 8  ........................................T....................... 55.6
       ............--.T..T............................................. 42.0

BE4 9  ........................................G....................... 100
```

Supplementary Fig. 1 Alignments of mutant sequences from founder mice carrying base-edited mutations. The sgRNA is underlined and the corresponding PAM sequence is shown in green. Within the editing window, the target nucleotides for base editing and nucleotides substituted by base editing are shown in bold black and blue, respectively. Bystanders are highlighted in red and deletions are indicated using a deletion symbol. WT, wild-type.

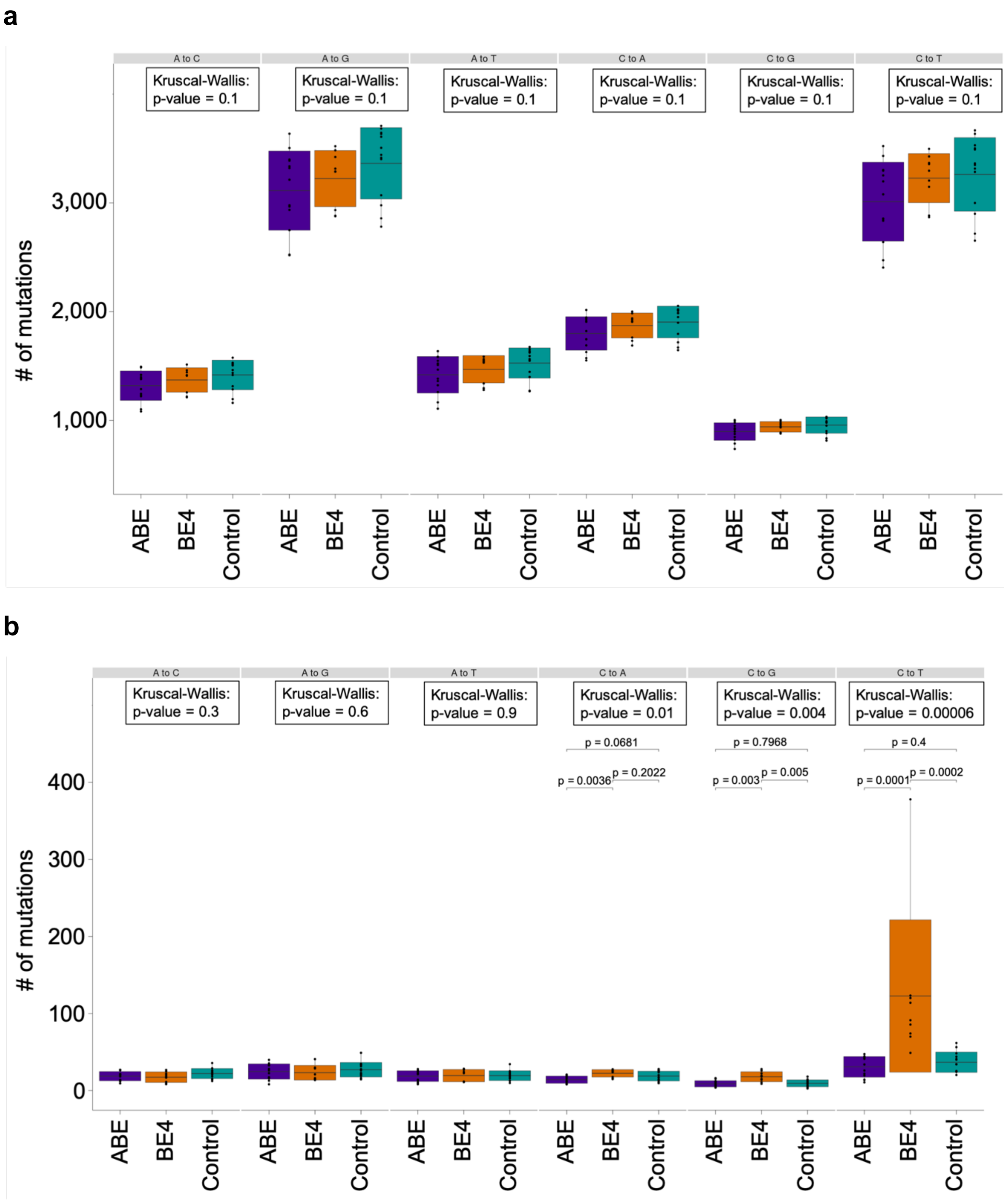

Supplementary Fig. 2 The frequencies of different types of total (a) and *de novo* SNVs (b) in individuals edited by the base editors and in the control group.
