# Supplementary material for: Cytosine base editor 4 but not adenine base editor generates off-target mutations in mouse embryos
Source: Commun Biol. 2020 Jan 9;3:19. doi: 10.1038/s42003-019-0745-3 (PMC6952419; doi:10.1038/s42003-019-0745-3)
Supplement: Supplementary file 2 — Description of Additional Supplementary Materials [file 42003_2019_745_MOESM2_ESM.pdf]

Description of additional supplementary materials

Supplementary Data 1. Raw read counts of WGS data

Supplementary Data 2. SNVs identified in each sample and de novo SNPs for the progeny

Supplementary Data 3. Indels dentified in each sample and de novo indels for the progeny

Supplementary Data 4. Location of the four predicted off-target sites overlapping with de novo SNVs. The samples harboring the same SNV are grouped into overlapping groups.

Supplementary Data 5. Characterization of de novo indels
